# Microwell-enhanced optical rapid antibiotic susceptibility testing of single bacteria

**DOI:** 10.1016/j.isci.2023.108268

**Published:** 2023-10-20

**Authors:** Ireneusz Rosłon, Aleksandre Japaridze, Stef Rodenhuis, Lieke Hamoen, Murali Krishna Ghatkesar, Peter Steeneken, Cees Dekker, Farbod Alijani

**Affiliations:** 1 Delft University of Technology, Mekelweg 2, Delft 2628 CD, the Netherlands; 2 SoundCell B.V., Raamweg 20D, The Hague 2596HL, the Netherlands

**Keywords:** Microbiology, Medical Microbiology

## Abstract

Bacteria that are resistant to antibiotics present an increasing burden on healthcare. To address this emerging crisis, novel rapid antibiotic susceptibility testing (AST) methods are eagerly needed. Here, we present an optical AST technique that can determine the bacterial viability within 1 h down to a resolution of single bacteria. The method is based on measuring intensity fluctuations of a reflected laser focused on a bacterium in reflective microwells. Using numerical simulations, we show that both refraction and absorption of light by the bacterium contribute to the observed signal. By administering antibiotics that kill the bacteria, we show that the variance of the detected fluctuations vanishes within 1 h, indicating the potential of this technique for rapid sensing of bacterial antibiotic susceptibility. We envisage the use of this method for massively parallelizable AST tests and fast detection of drug-resistant pathogens.

## Highlights

• A single-cell optical antibiotic screening technique is developed
• Using microwells, single bacteria are trapped in the laser spot
• Laser intensity fluctuations are correlated with bacterial resistance
• Signal fluctuations diminish within 1 h when antibiotics kill the bacteria

## Graphical abstract

**Figure:**
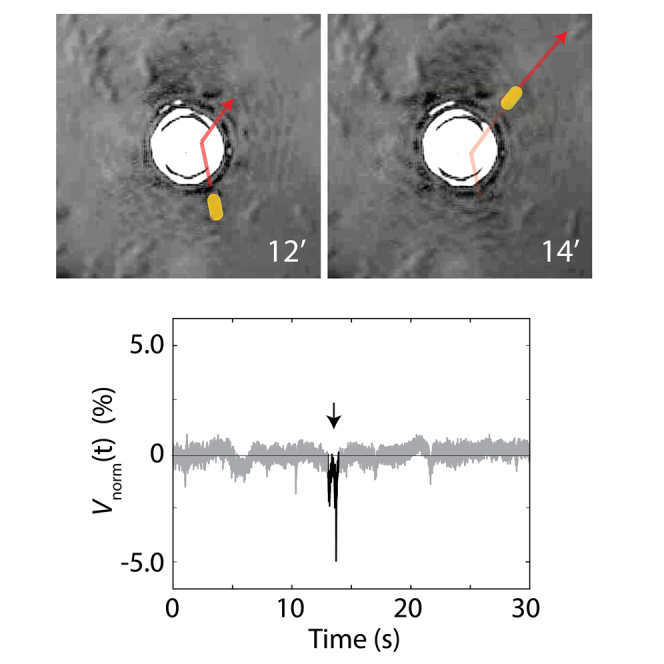

## Introduction

Antibiotic resistance is a major challenge for mankind.^1^ Most common antibiotic susceptibility testing (AST) methods are based on the detection of the growth rate of the pathogenic organisms or changes in the concentration of a marker molecule in solution.^2^ In a clinical setting, the commonly adapted AST methods include measurement of the turbidity of the growth solution, its carbon dioxide content, or the diameter of the inhibition zone around an antibiotic disk.^3^ These methods are the “gold standards” in the clinic and have been used for over half of a century for their reliable and reproducible determination of minimum inhibitory concentrations (MICs) of an antibiotic. However, these conventional methods are slow, due to their dependency on the growth rate of the pathogenic microorganism. As a result, AST methods typically require between 16 and 48 h before any results can be obtained,^4^ and for slowly growing pathogens, this waiting time may take up to weeks.^5^ The slow growth also causes delays in determining the pathogen identity, typically done by MALDI-TOF mass spectrometry,^6^ and therefore in prescribing the right antibiotic to patients. Numerous studies are being conducted to shorten the time between isolation of the pathogen and performing a rapid AST.^7^^,^^8^ The target is to prescribe antibiotics on the same day as the diagnosis, which essentially means that AST should be performed within 8 h or less^9^ and ideally even within an hour.

Various new methods have shown potential to obtain AST results faster than traditional methods.^10^^,^^11^^,^^12^ These methods include full genome screening, microfluidic-based assays,^8^^,^^13^ optical methods,^14^ and nanomotion-based techniques.^15^^,^^16^ Many of these emerging technologies obtained positive results in a laboratory setting but face issues in clinical practice, where low cost and high throughput are key factors. Among them optical microfluidic devices are good candidates for fast AST platforms that offer results within hours.^17^^,^^18^

Optical measurements typically involve a multichannel device where bacteria in growth medium are subjected to various antibiotics at different concentrations. The readout often requires a marker that reacts to a change in, for example, pH or CO2 concentration to indicate bacterial growth.^19^^,^^20^ Recent reports indicate that light scattering from bacteria correlates with readout from fluorescent markers, which would allow for AST without the use of a such marker.^21^ Alternative label-free optical microscopy methods exist too, such as those that count cell division events as an indicator of cellular growth.^22^^,^^23^ This approach has also been successfully used to detect morphology changes that indicate resistant response at a single-cell level.^24^ Furthermore, some of these techniques show the capability of performing identification and possibly working with mixed samples.^25^ In addition, optical phenotypic monitoring, which involves the detection of the motion of (groups of) motile pathogens, is interesting for its potential of performing AST within a few hours via tracing the changes in laser intensity when bacteria swim through a laser beam.^14^ Although promising, the dependency of all these techniques to bacterial concentrations is a problem, because for low bacterial concentrations, long measurement times are often required to obtain sufficient statistics for determining the susceptibility of a microorganism to an antibiotic. Thus, methods are needed that enhance the likelihood of bacteria crossing the laser light.

Here, we present a new reflectometric readout technique that performs AST on weakly trapped motile bacteria, which greatly enhances the sensitivity of measurements, even to the level of detection of single bacteria. The technique detects bacterial motility via intensity variations in the laser light when a bacterium crosses the path of the laser beam that is reflected from the silicon surface. Recently, we used suspended graphene drums to detect the nanomechanical motion of single bacteria adhered to its surface.^16^ Such technique is based on the transduction of the mechanical nanomotion of the adhered bacterium through a mechanical lever.^15^ Furthermore, rapid AST tests are under development that are based on molecular biology and make use of microfluidics, electrochemistry, or genomics.^12^ Here, however, we present a much simpler principle by showing that bacteria viability can be detected solely by optical means. By patterning the surface with microwells that physically localize the bacteria within the laser focus, we increase the crossing event frequency. We expect that bacteria encountering the edge of the microwell will experience a resistance that induces tumbling or alters the path such that the bacteria follow the edge. In either case, the position of the bacterium would be restricted to the area of the microwell, and a weak trapping is realized. We demonstrate that the signal is significantly enhanced when cells are measured in proximity of a reflective surface and show that our reflectometric readout system can be used for fast detection of the susceptibility of motile bacteria to antibiotics, opening a new route for rapid AST.

## Results

### Optical detection of single motile bacteria

**Figure 1:**
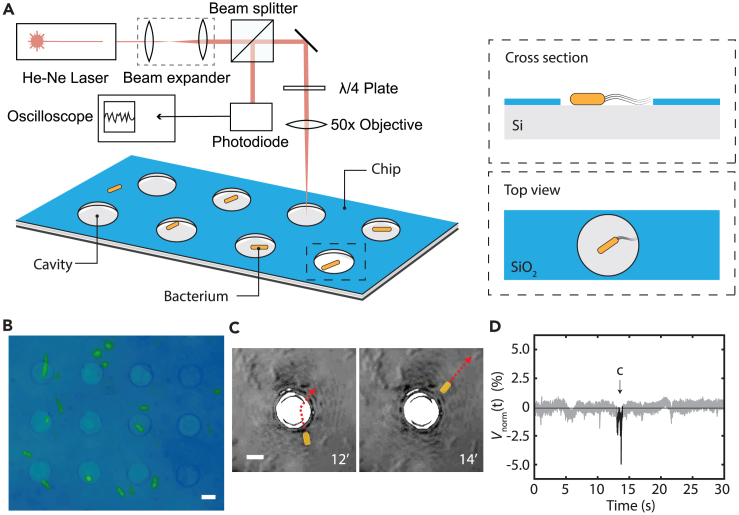
Laser detection method for motile bacteria (A) Schematic illustration of interferometric readout system used to localize bacteria in pre-patterned Si/SiO_2_ microwells with 8 μm diameter and 285 nm depth. Bacteria on the patterned surface experience trapping and stay longer inside the cavities than outside. (B) Optical image of the fabricated microwells with fluorescent labeled bacteria; scale bar: 5 μm. (C) *In-situ* false-colored optical microscope image of microwells with an *E. coli* cell in lysogeny broth (LB) suspension crossing laser focused on microwell. *E. coli* swimming path through microwell is indicated by a dotted line. Scale bar: 5 μm. (D) Drop in the detected signal during the bacterium crossing the laser path depicted on panel C (signal highlighted in black). Measurement performed at *E. coli* concentration with OD = 0.05.

When an *Escherichia coli* bacterium passed through the laser focus, a sudden decrease in $V_{\text{norm}}\left(t\right)$ was recorded. Figures 1C and 1D as well as Video S1 present an example, where we simultaneously acquired the signal intensity and performed optical tracking of a cell during such an event. We observed fluctuations in the readout signal when a bacterium crossed the laser focus from the bottom to the upper right. Such fluctuations were not observed when bacteria were absent (see Figure S1). In the presence of motile bacteria, the fluctuations amounted up to a 10% of the total light intensity incident on the photodiode. We shall note that Video S1 is a sped up version (3.3 times) of the full recording from which an excerpt is shown in Figures 1C and 1D.

Video S1. Laser intensity fluctuations when a single bacterium passes the laser beam, related to Figure 1

**Figure 2:**
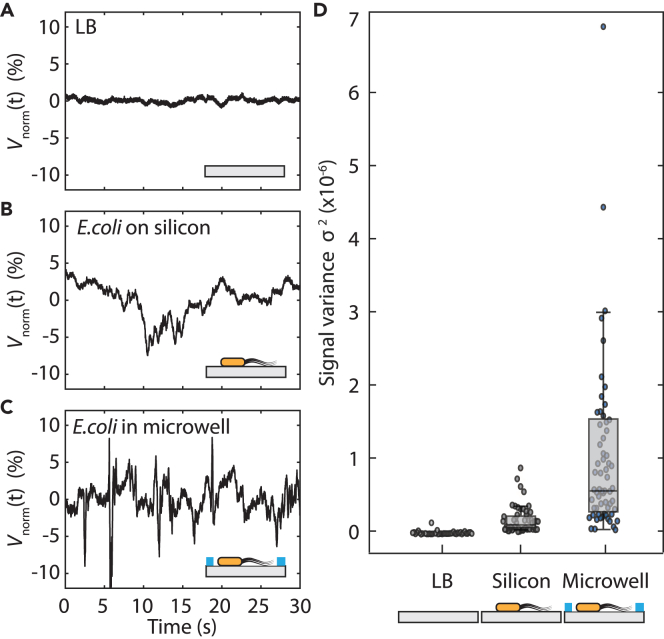
Experiments on silicon surface and in microwells (A–C) Typical signals recorded in three cases: (A) a control measurement on flat silicon with LB without bacteria, (B) on flat silicon surface with LB containing bacteria, and (C) on a microwell containing bacteria in LB. (D) Signal variance for these three cases: on reflective bare silicon with LB (n=43), same in the presence of bacteria (n=54), and on bacteria in micro-patterned wells (n=67). Signal fluctuations appear only when bacteria are present, and signals are prolonged when the surface is patterned with microwells.

To enhance the frequency of events, we introduced micro patterned silicon substrates, where we used microwells (see Figure 1A) to localize the bacteria. Such microwells are known to be able to trap the bacteria for prolonged time^26^ and can thus be used to maintain them in close vicinity of both the laser focus and the silicon surface without impeding their motility. While focusing the laser onto a microwell, we observed that the signal appeared in prolonged “bursts”: periods of increased fluctuations that were followed by a period of relative rest. As a result, individual traces in this case showed extended fluctuations (see Figure 2C as an example). We performed the three different experiments in lysogeny broth (LB) growth medium: without bacteria on bare silicon (as a control experiment), with bacteria on bare silicon, and with bacteria on micro-patterned cavities. Typical traces at *E. coli* concentration with optical density OD = 0.2 are shown in Figures 2A–2C. A statistical analysis of the variance of such traces is shown in Figure 2D.

When we performed the experiment on flat silicon, we noted that bacteria crossed the laser focus only rarely during the 30′ observation windows, and traces for bacteria on bare silicon accordingly showed a variance that is only slightly higher than that in the control experiment. Indeed, in Figure 2B, similar to Figures 1C and 1D, we saw a single crossing event where only a spike in the intensity signal could be observed. The measurements on microwell substrates, however, showed on average a significantly higher variance. The probability for detecting laser intensity variations due to bacteria crossing the laser path was thus significantly enhanced by performing the experiment in the microwells where the bacteria got trapped. The localization of the bacteria inside the microwells was further confirmed by microscopic imaging on transparent PDMS samples with the same geometry as the silicon (see Figure S2), in which we observed a 50% higher probability of imaging a bacterium inside a well than outside of it. Furthermore, we performed time-lapse microscopy imaging to determine the fraction of motile bacteria in the sample, which confirmed that a large portion of bacteria are actively swimming in the sample (See Figure S3). In addition, we investigated the influence of bacteria quantity on the observed signal fluctuations. To that end, we performed experiments where we observed that the signal fluctuations increase with the increase in cell density, which is the result of more cells passing through the laser beam (see Figure S4).

### Dependence of readout signal on bacterium size

The finding that signal fluctuations are due to bacteria crossing the laser path suggests that the strength of the readout signal could be dependent on the size of the bacteria. Because the laser beam is larger than the bacterium diameter, changing the bacterium shape or size can be expected to cause a different light refraction and absorption by the cell. In order to test this hypothesis, we measured the signal of shape- and size-manipulated *E. coli* cells. We grew the bacteria in the presence of low doses of A22^27^ or Cephalexin,^28^ which changed the bacterial cells into spherical and tubular shapes, respectively.

**Figure 3:**
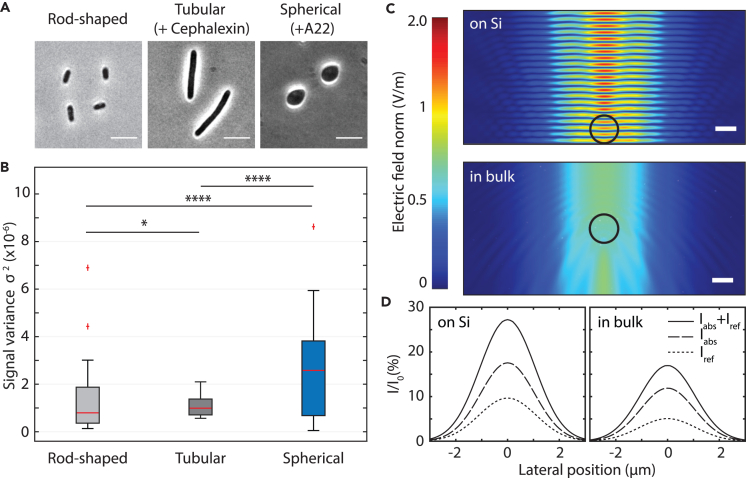
Cell size and position determine the amount of light that is attenuated (A) Phase contrast images of the three different sized *E. coli* cells used in our experiments. Scale bar: 5 μm. (B) Microwell measurements were performed on bacteria with different cell sizes: normal rod-shaped cells (3.4 micron in length), long tubular cells (10 micron in length), and spherical cells (4.2 micron diameter). Boxplot whiskers extend to maximum 1.5 times the inter-quartile distance, and outliers are indicated with crosses. Red horizontal line represents the median values, and significance is expressed using the asterisk convention. (C) Simulated electric field amplitude *E* of a focused 633 nm laser beam around an *E. coli* cell (indicated by a black circle) on a perfectly reflective silicon surface (top graph) and freely suspended in LB medium (bottom graph). The laser is incident from the top of the plot and the silicon surface is at the bottom of the top plot. Scale bar equals 1 μm and laser beam waist equals 4 μm, similar to experimental conditions. (D) Fraction of the incident laser power that is attenuated ($I_{\text{abs}}+I_{\text{ref}}$) by absorption ($I_{\text{abs}}$) and refraction ($I_{\text{ref}}$) as a function of the lateral position of the cell, in cases of both on the reflective silicon surface (left) and freely suspended in the growth medium (right).

Figures 3A and 3B compare the data for bacteria with different sizes. Normal rod-shaped *E. coli* cells had a length of 3.4 ± 0.6 micron (n=51) and a width of 1.0 ± 0.1 micron, whereas spherical A22-exposed cells had a diameter of 4.2 ± 0.6 micron (n=34). The cells that were exposed to Cephalexin grew along the longitudinal axis, forming tubular shapes with a length of 10 ± 2 (n=43) microns. As expected, changes in cell shape influenced the observed signal fluctuations, with larger cells generating larger signal fluctuations (See Figure S6 for more details).

### The role of position, absorption, and refraction in signal detection

Light intensity fluctuations can be attributed to two main sources: first, the absorption of the laser light by a bacterium and second, refraction of light at the boundary of the bacterium, caused by the difference in the refractive indices of the cell and the surrounding medium. Typical values of the refractive index for *E. coli* are 1.39±0.05, whereas values for LB medium have been reported as 1.335±0.03.^29^^,^^30^^,^^31^ Light traveling through a bacterium is absorbed more than in the surrounding liquid, a property that is typically used in cell counting experiments by optical density (OD) measurements.^32^^,^^33^^,^^34^ For *E. coli* cells we used an attenuation coefficient of $\mu =1.1\times 10^{5}$ $m^{-1}$ (see STAR Methods). Using these estimates, we performed COMSOL finite element simulations of Maxwell equations to explore the influence of a bacterium on the optical field and to find what portion of light is attenuated by a single bacterium passing through a focused laser beam (see also Figure S5). In these simulations, the distortion and intensity change of a Gaussian beam with a waist diameter of 4 μm was calculated in LB medium, both without and in the presence of an *E. coli* bacterium.

Figure 3C shows the electric field amplitude *E* for both the case where the bacterium is near the silicon surface and for the case that it is far from it. Simulations were performed in 2D, and the cell is represented by a black circle. The interference between incident and reflected light waves results in a prominent standing wave near the silicon surface. In the absence of the silicon substrate there is no standing wave, and the refraction caused by the bacterium can be observed clearly. These calculations were repeated for various positions of the cell relative to the laser focal position, to simulate a bacterium swimming through the center of the beam (see also Figure S5). Furthermore, we calculated the absorption of the electric field $I_{\text{abs}}$ by the bacterium for all lateral positions by integrating the power loss *p* over the cell area *A*, $I_{\text{abs}}={∯}_{A}pdA$, as shown in Figure 3D as a percentage of the total incoming optical power $I_{0}$. We also computed the amount of light $I_{\text{ref}}$ that is refracted by an angle greater than 45° (the limit due to the numerical aperture of the lens), i.e., the light not returning to the detector—again as a function of the lateral position of the bacterium. Far outside of the laser beam, obviously, the bacterium does not absorb nor refract light. In the center of the laser beam though, up to 18% of the incoming light is absorbed and up to 10% is refracted if the bacterium is on the silicon surface, which indicates that both absorption and refraction by the cells play a role. These numbers are similar to the value in experiments, where typical oscillations are 10% and the highest peak-to-peak variations that we observed were 20% of the total signal amplitude. Notably, in most experiments, the cells did not cross the beam exactly in the center of the laser focus, and hence, the experimental values are lower than simulated.

The bacteria close to the silicon surface yielded a signal that is about twice higher than that of bacteria that were swimming freely in bulk LB (see Figure 3D). Because the laser beam is focused with a 0.55 NA objective to a 4 μm spot, creating of conical bundle with a $46^{\circ }$ angle, we are mostly sensitive to bacteria close to the focal point. The light beam quickly spreads wider away from the surface. For example, at a height of 10 μm away from the surface, the light beam cross-section is already 12.7 μm, i.e., about 10 times larger than the cross-section of a typical bacterium, and the signal from a bacterium crossing far away from the focal point is reduced by 10-fold. Accordingly, bacteria need to be close to the focal point to be detected. Guiding or trapping bacteria near the surface and laser focus can thus improve the readout signal in addition to increasing the event frequency of bacteria passing the laser light.

### Antibiotic susceptibility testing

Finally, we explored if this method can be applied for testing the efficacy of antibiotics. We compared the signal of live bacteria on bare silicon and patterned microwells with the signal of the bacteria after exposure to various antibiotics. We tested chloramphenicol, an antibiotic that blocks protein synthesis,^35^ and ciprofloxacin, an antibiotic that blocks the activity of DNA gyrases.^36^ Importantly, these antibiotics at low concentration and short exposure times do not affect the morphology, the size and shape, of the bacteria (Figure S7).

**Figure 4:**
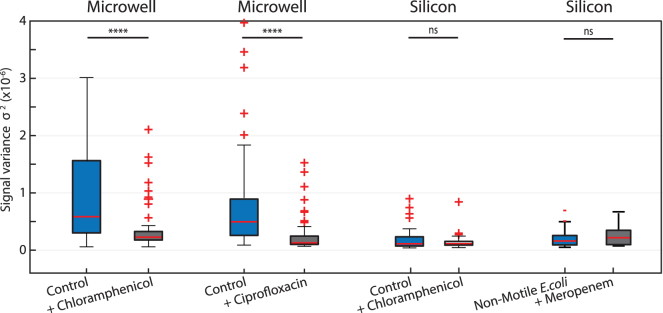
Effect of antibiotics on the observed signal amplitude Signals before and after administering various antibiotics; 1 h after administering chloramphenicol (34 μg/mL), 1.5 h after administering meropenem (50 μg/mL), and 3 h after administering ciprofloxacin (20 μg/mL). On the etched microwells (*n* = 67), after administering antibiotics, chloramphenicol (*n* = 67) or ciprofloxacin (*n* = 200), a significant drop of the initial signal ($p<10^{-5}$, ∗∗∗∗) can be observed for susceptible bacteria. For measurements on bare silicon (*n* = 54), no significant difference (p=0.94, ns) was measured after exposure to the antibiotic (*n* = 54). Also, *E. coli motAB* non-motile cells were tested, and no statistical difference between signal from non-motile (n=71) and antibiotic-treated cells (n=82) was observed. Boxplot whiskers extend to maximum 1.5 times the interquartile distance, and outliers are indicated with crosses. Red horizontal line represents the median values. Measurements are compared using a two-tailed Wilcoxon rank-sum test.

Figure 4 shows the signal variances before and after administering the antibiotic for both cases. One hour after the addition of antibiotics, there was a significant drop in the signal for both antibiotics. For the data on a silicon surface, however, no significant change could be observed after addition of the chloramphenicol.

**Figure 5:**
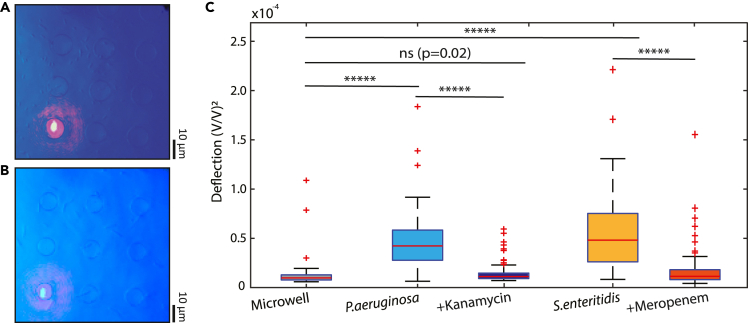
Antibiotic susceptibility of clinical isolates (A) Optical image of microwells in the presence of *P. aeruginosa L1262* strains. (B) Optical images of microwells in the presence of *S. enteritidis S1400* strains. (C) Signals before and after administering various antibiotics to the clinical isolates and comparison to empty microwells; 1 h after administering kanamycin (25 μg/mL) to the *P. aeruginosa* and 3 h after meropenem (10 μg/mL) to *S. enteritidis*. After administering the antibiotics, kanamycin (*n* = 100) or meropenem (*n* = 62), a significant drop of the initial signal ($p<10^{-5}$, ∗∗∗∗) could be observed for susceptible bacteria (*n* = 100). For measurements on empty microwells (*n* = 53) no significant difference (p=0.02, ns) was measured compared with antibiotic-exposed samples. Red horizontal line represents the median values. Measurements are compared using a two-tailed Wilcoxon rank-sum test. The red circle in 5A and 5B show the laser spot in a microwell.

To test the efficacy of the technique in detecting antibiotic resistance, we also performed an additional experiment on *E. coli* with *KanR* resistance gene.^37^ We exposed these resistant cells to kanamycin,^38^ an antibiotic that inhibits protein synthesis, but we did, as expected, not observe a change in the variance of the signal after administering the antibiotic (see Figure S8). Even after several hours of incubation, the signal stayed unchanged, demonstrating that the technique is able to demonstrate not only susceptibility to antibiotics but also the resistance of bacteria against them. The technique was also applicable to detecting antibiotic susceptibility of motile clinical isolates of *Pseudomonas aeruginosa* and *Salmonella enteritidis*. These motile isolates (see Figure 5) were exposed to kanamycin and meropenem antibiotics, respectively. After just few hours of exposure, there was a clear drop in the signal, which was non-distinguishable from the background microwell signal. These results were also in full agreement with AST performed on the same bacteria by the disk diffusion method (See Figure S9).

In a recently published study, widefield optical microscopy was employed for the detection of movement of single bacteria.^39^ This study reported observation of motion even for non-motile bacterial species such as *Staphylococcus aureus*, which diminished after treatment with antibiotic. We tried to reproduce these surprising findings, so we tested *E. coli* motAB non-motile cells, but we did not observe a significant change in signal amplitude before and after antibiotic treatment (see Figure 4). In fact, we observed signals that were of the same magnitude as background (see Figure S10). We note that we performed a Wilcoxon rank-sum test in order to compare the two non-normal distributions, whereas this recent study^39^ performed a Student’s t test. This might explain the difference in the conclusions of the works.

## Discussion

We presented an optical detection technique to measure the viability of single motile bacterial cells. Our method is based on the fluctuations of a laser signal when bacteria run through its focal plane. We observed that signal fluctuations depend on morphology, movement, and quantity of bacteria cells. Therefore, to disentangle these effects from one another, we performed independent measurements where we varied only one parameter at a time. To extend the time during which a bacterium motility can be measured in the laser spot, we introduced microwells in the silicon surface with 285 nm depth and 8 μm diameter. Because the bacteria are trapped at these predetermined microwell spots, the bacteria stay longer near the laser spot (Figure 1A), and more events can be observed during the measurement window, i.e., larger signals are collected. The cavity dimensions are chosen to be comparable with the size of bacteria. A relatively shallow cavity depth minimizes optical aberrations that would be introduced by the sidewalls. To yet further increase the throughput of this method, a more elaborate chip design can be conceived. One could, for example, guide bacteria toward the laser focus by channels or mazes,^40^ which would allow samples at lower concentrations to be used for detection. Optimization of the trap depth might also aid measurements—see for example Figure S10 where the duration of trapping events is prolonged—although too deep traps will impact readout quality adversely and might limit the natural motility of the bacteria.

Next to our experimental observations, we performed numerical studies and concluded that the variations in the reflected signal can be explained by a combination of refraction and absorption of the laser light by the *E. coli* bacteria. Peak variations in signal during experiments (up to 20%) were of comparable magnitude as the maximum variations that were calculated from the simulations (maximum 28%). Our finite-element simulations showed that bacterial motion resulted in larger signal fluctuations near a reflective surface than in the free volume. The simulations provide a better understanding of the optimal conditions for optical detection.

The detected signal in measurements of the bacteria described here is directly linked to the motility of the pathogens, which vanishes upon exposure to antibiotics. The single-cell method presented here requires fewer cells for the analysis, thus speeding up AST procedure. Moreover, the method can be scaled up, such that it allows assessing whether the strain is resistant or sensitive based on the statistics obtained from hundreds of single-cell measurements. We believe that the high-speed nature of our technique will be helpful for developing rapid diagnostic tools for detection of motile pathogens. For example, in urinary tract infections by *E. coli* (which accounts to 75% of infections),^41^ we envisage our technique to be highly efficient. To be suitable for everyday clinical practice, however, the technique requires improvements in terms of convenience and throughput. Most notably, a setup capable of obtaining measurement in parallel on multiple points would be required to scan through various antibiotics and antibiotic concentration. Yet, it is important to highlight that we could detect the susceptibility and resistance to a single antibiotic in less than an hour, which is significantly quicker than existing detection techniques based on growth rate of bacteria that typically take days.^42^ We are confident that the current results provide a good base to further accelerate the development of next-generation AST tests.

### Limitations of the study

The technique described in this work is ineffective for non-motile pathogens. Therefore, in case of an infection with a non-motile pathogen, alternative methods must be used for AST.

## STAR★Methods

### Key resources table

**Table:** 

REAGENT or RESOURCE	SOURCE	IDENTIFIER
**Bacterial strains**		

*E.coli* MG1655(+IS1)	Tu Dleft, Bionanoscience Dept.	Hypermotile 7740
*E.coli* MG1655(KanR)	Tu Dleft, Bionanoscience Dept.	BN2830
*P.aeruginosa* clinical isolate	Tu Dleft, Bionanoscience Dept.	L1262
*S.enteritidis* clinical isolate	Reinier Haga MDC, Delft, NL	S1400

**Software and algorithms**		

MATLAB2022	Mathworks	https://www.mathworks.com/products/mat lab.html

### Resource availability

#### Lead contact

Subsequent inquiries and requests should be sent to and will be fulfilled by the the corresponding author, F. Alijani (f.alijani@tudelft.nl).

#### Materials availability

This study generated no unique materials.

### Experimental model and study participant details

The bacterial strains used in this study included: *E.coli* MG1655(+IS1) (Hypermotile 7740), *E.coli* MG1655(KanR), clinical isolate of *P.aeruginosa* as well as clinical isolate of *S.enteritidis*. Among these species, *E. coli* strains and *P.aeruginosa* were obtained from department of Bionanoscience at TU Delft, and *S.enteritidis* were obtained from Reinier Haga MDC in Delft, the Netherlands.

### Method details

#### Sample preparation

All experiments we performed on MG1655(+IS1) *E. coli* cells, described earlier.^43^ Experiments with Kanamycin resistant *E.coli* cells were performed on MG1655(*kanR*) cells described earlier.^16^ All bacterial cells, were grown in LB medium overnight at $30^{\circ }$ C to reach the late exponential phase. The next day before performing experiment, the culture was refreshed (1:100 volume) for 2.5 hours on fresh LB medium at $30^{\circ }$ C reach an optical density (OD600) OD=0.2. The chamber was filled with the solution at this concentration, unless stated otherwise, and left for 15 minutes horizontal position to deposit the bacteria on the surface. For experiments where antibiotics were used, antibiotics were dissolved in LB and incubated with bacteria for 1h. Chloramphenicol was used at 34μmg/ml, Ciprofloxacin at 20μg/ml, Kanamycin at 25μg/ml and Meropenem at 10 or 50μg/ml final concentration. An optical microscope (Keyence VHX-7000) was used to inspect the sample. The chamber was placed in the interferometric setup that was equipped with Attocube ECSx5050 nano positioners that allow automated scanning. The motion of the bacterium caused changes in the optical path, that were monitored by a photodiode and an oscilloscope (Rohde & Schwarz RTB2004). At each measured point on the substrate, a trace was recorded for 30 seconds with 50’000 data points. The setup was programmed to run through 200 measurement points per condition but would terminate sooner if the wells were no longer in focus. This was verified by monitoring the absolute signal amplitude, and terminating the measurement if a large decrease in reflected light amplitude occurred. The measurements were performed in an air-conditioned room with a temperature of $21^{\circ }$ C. The substrates were either 5x5 mm^2^ silicon chips, or 5x5 mm^2^ silicon chips with a 285 nm layer of silicon oxide. The latter were patterned with circular cavities by a reactive ion etch, where silicon acted as a stop layer, creating cavities with a diameter of 8 μm, described earlier.^16^

#### Optical detection

The experiments were performed on silicon samples that were placed inside a cuvette containing motile MG1655 *E.coli* in LB medium. We recorded the intensity of the reflected 633 nm He-Ne red laser light (see STAR Methods), that was focused on the silicon surface to a spot of 4 μm in diameter, using a laser reflectometry setup as depicted in Figure 1. The crossing of a bacterium through the focal region could be determined from the modulation of the intensity of the light that returned to the photodiode. Individual traces are normalized relative to their mean intensity, $V_{\text{norm}}\left(t\right)=\left(V\left(t\right)/<V\left(t\right)>\right)-1$, where the brackets stand for the time average. The reflected laser intensity V(t) was measured for 30’, and the signal variance $\sigma^{2}=<{\left(V_{\text{norm}}\left(t\right)\right)}^{2}>$ was used as a metric to compare various traces.

#### Bacterial shape manipulation

In order to grow the *E.coli* cells into spherical shapes, low doses of the A22 drug were added to the to LB. On the day of the experiment, the cell culture was refreshed (1:100 volume) in the presence of A22 drug (5μg/ml final concentration) for 1.5 hours on fresh LB medium at $30^{\circ }$ C reach an optical density (OD600) between OD=0.2–0.3. A22 inhibits the MreB polymerization, thereby disrupting the typical rod shape of *E. coli*.^27^ These spherical cells remain physiologically active and can replicate and divide.^44^^,^^45^ In order to grow the cells into tubular shapes, low doses of cephalexin drug (25μg/ml final) were added to the to LB and cells were grown for 1 hours on fresh LB medium at $30^{\circ }$ C. Cephalexin blocks cell division but allows cells to grow in length.^28^

#### Optical microscopy

To measure the sizes of *E.coli* cells we used Nikon Ti-E microscope with a 100X CFI Plan Apo Lambda Oil objective with an NA of 1.45 equipped with a phase ring. Images were captured by Andor Zyla USB3.0 CMOS Camera.

#### Laser interferometry

A red laser ($\lambda_{\text{red}}=632.8$ nm) focused with a 4 μm spot size on the sample was used for detection of the amplitude of the cell motion, where the position-dependent optical absorption of the cell results in an intensity modulation of the reflected red laser light, that was detected by a photodiode.^46^ The incident red laser power was 3 mW.

#### Calculation of linear attenuation coefficient

The optical density (OD) of a sample is defined as the logarithm of the ratio between the incident and transmitted laser power, that is: $OD=log_{10}\left(I_{1}/I_{0}\right)$. This means that at OD=1, a fraction x=0.1 of the incident light is transmitted. A measurement of OD=1 corresponds to approximately $10^{9}$ bacteria /mL in a 1 cm cuvette.^47^ The fraction of light *x* that is transmitted by a single bacterium can thus be expressed as $x={\left(I_{1}/I_{0}\right)}^{\sigma_{c}/\sigma_{t}}$, where $\sigma_{c}$ is the physical cross section of the cuvette and $\sigma_{t}$ is the total cross section of *n* bacterial cells in suspension with each a physical cross section σ, i.e. $\sigma_{t}=n\sigma$. We wish to compute the linear attenuation coefficient μ, which relates the transmitted laser power to the distance *d* travelled through a bacterium by the following expression: $I\left(d\right)=I_{0}\cdot e^{-\mu d}$. This can be rewritten into μ=−ln(x)/d. From the measured physical cross section of a single bacterium (A≈1×2μ $m^{2}$)^48^ and the cross section of the cuvette (A=1 ${\text{cm}}^{2}$), we find that a single bacterium absorbs around x=11% of the incoming light and an attenuation coefficient of $\mu =-\ln\left(x\right)/d=1.1\times 10^{5}$ $m^{-1}$ for *E.coli* cells with average diameter d=1μ m.

#### Data processing

The signal obtained from the photodiode voltage due to the variations in reflected intensity of the red laser is recorded by an oscilloscope. The time trace of the photodiode voltage $V_{\text{pd}}$ (t) was normalized by division over its average, $V_{\text{norm}}\left(t\right)=V_{\text{pd}}\left(t\right)/<V_{\text{pd}}\left(t\right)>$, after which a linear fit was subtracted from the data to eliminate the effects of drift during the measurements.

### Quantification and statistical analysis

Since the data reported in the paper are not normally distributed, we relied on non-parametric tests for statistics. We represent the median and quartiles of data in boxplots, in accordance with the use of non-parametric tests. We use a rank sum test for comparison between measurement sets. We used MATLAB’s built-in functions for statistical analysis. All statistical tests were two-sided. On all figures, the following conventions are used: not significant (NS) 0.05 < P, ∗0.01 < P < 0.05, ∗∗0.001 < P < 0.01, ∗∗∗0.0001 < P < 0.001, ∗∗∗∗P < 0.0001. We report a significant difference in results if P < 0.01.

## Data Availability

All original data has been deposited at the 4TU Repository (https://doi.org/10.4121/34e0bc18-9438-4b32-b6d0-84a9d65f22d7.v1) and are publicly available as of the date of publication. DOI is listed in the key resources table. All original code is deposited at the 4TU Repository (https://doi.org/10.4121/34e0bc18-9438-4b32-b6d0-84a9d65f22d7.v1).
