# Supplementary material for: Microwell-enhanced optical rapid antibiotic susceptibility testing of single bacteria
Source: iScience. 2023 Oct 20;26(11):108268. doi: 10.1016/j.isci.2023.108268 (PMC10654606; doi:10.1016/j.isci.2023.108268)
Supplement: Document S1. Figures S1–S10 and Table S1 [file mmc1.pdf]

iScience, Volume *26*

Supplemental information

Microwell-enhanced optical rapid antibiotic susceptibility testing of single bacteria

Ireneusz Rosłon, Aleksandre Japaridze, Stef Rodenhuis, Lieke Hamoen, Murali Krishna Ghatkesar, Peter Steeneken, Cees Dekker, and Farbod Alijani

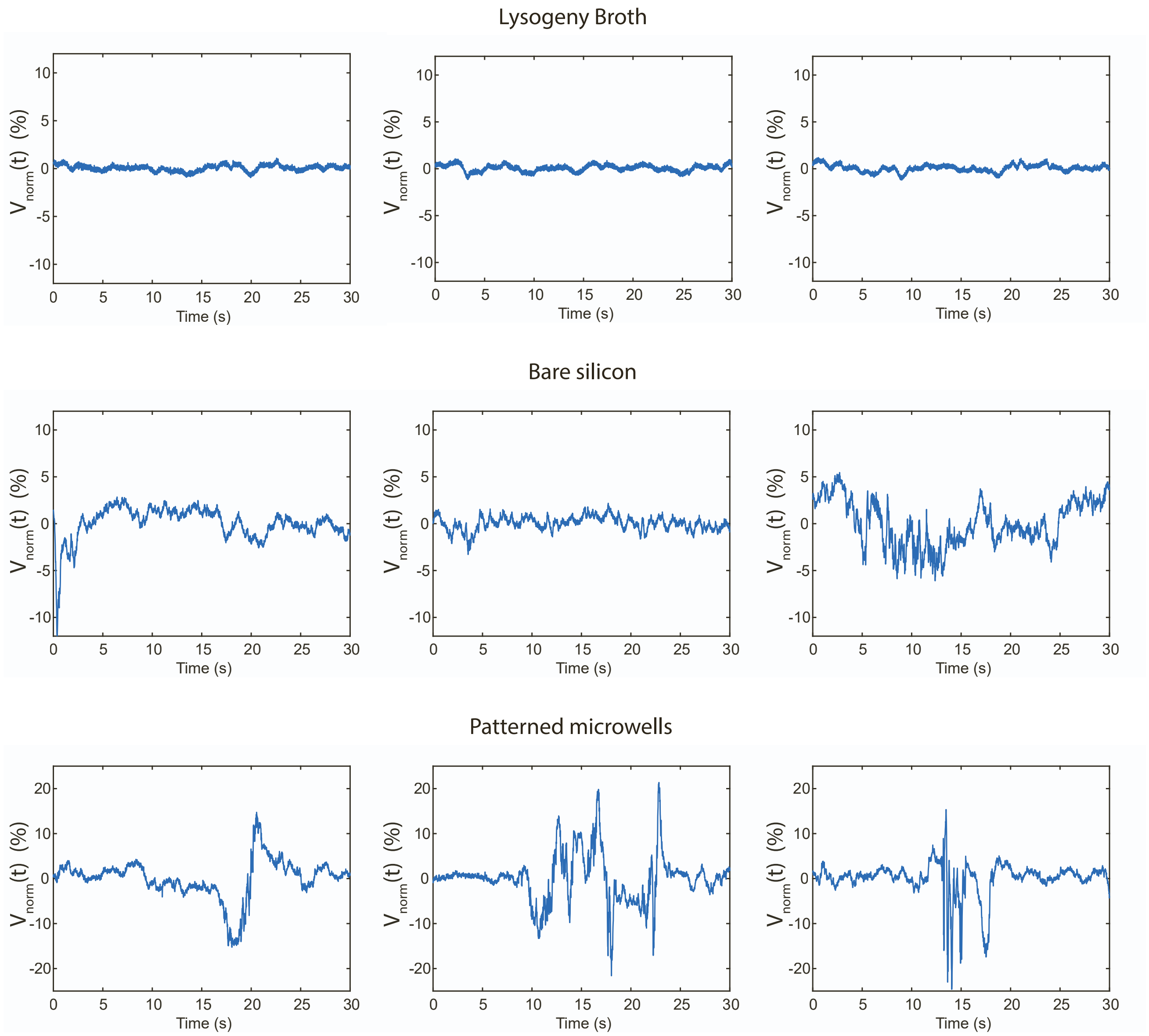

FIG. S1. Impact of substrate on observed signal, related to Figure 1. Each graph shows a measurement performed on single position in the absence of bacteria (top), in the presence of bacteria and on bare silicon (middle) or inside a microwell (bottom), during 30 seconds.

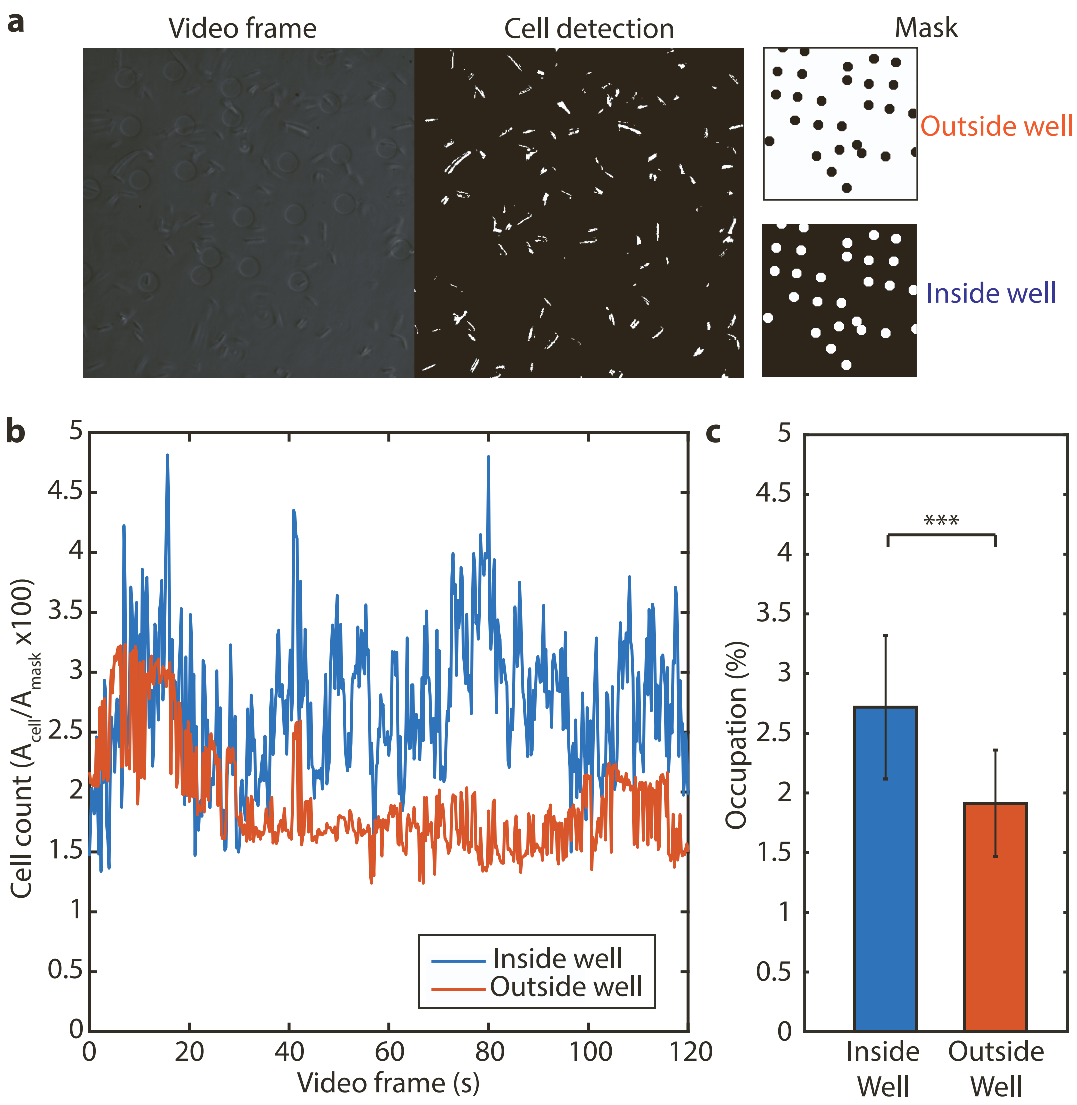

FIG. S2. Occupation of 285nm deep PDMS wells with *E.coli* cells, related to Figure 2. a) A raw video frame and the result of the cell detection algorithm used for analysis. The samples were placed under a Nikon Ti-E microscope with a 100X CFI Plan Apo Lambda Oil objective with an NA of 1.45 equipped with a phase ring. Videos of 120s were recorded to observe the residence of the cells. The videos were analyzed using a MATLAB script to track the cells and compare occupation of areas inside and outside of the wells. The areas where cells are present are marked white. Then, a mask is applied to find if a cell is inside or outside of a well. For each movie frame, we calculated the cell count normalized by the mask area, $A_{\mathrm{cell}}/A_{\mathrm{mask}}$, both inside and outside of the wells. b) The percentage of the total area that is occupied by cells is calculated both inside and outside of wells for the entire length of the video. c) The occupation of wells is approximately 50% higher than of the surrounding area.

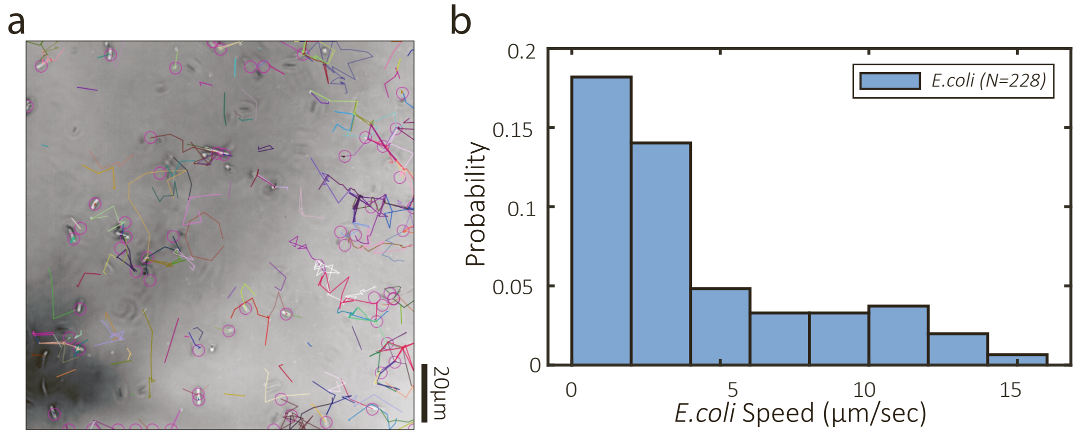

FIG. S3. Time-lapse microscopy imaging, related to Figure 2. a) Optical image of bacteria with overlayed swimming traces (colored tracks). b) Average swimming speed probability for *E.coli* (N=228). The mean speed of bacteria is tracked with TrackMate plugin [1].

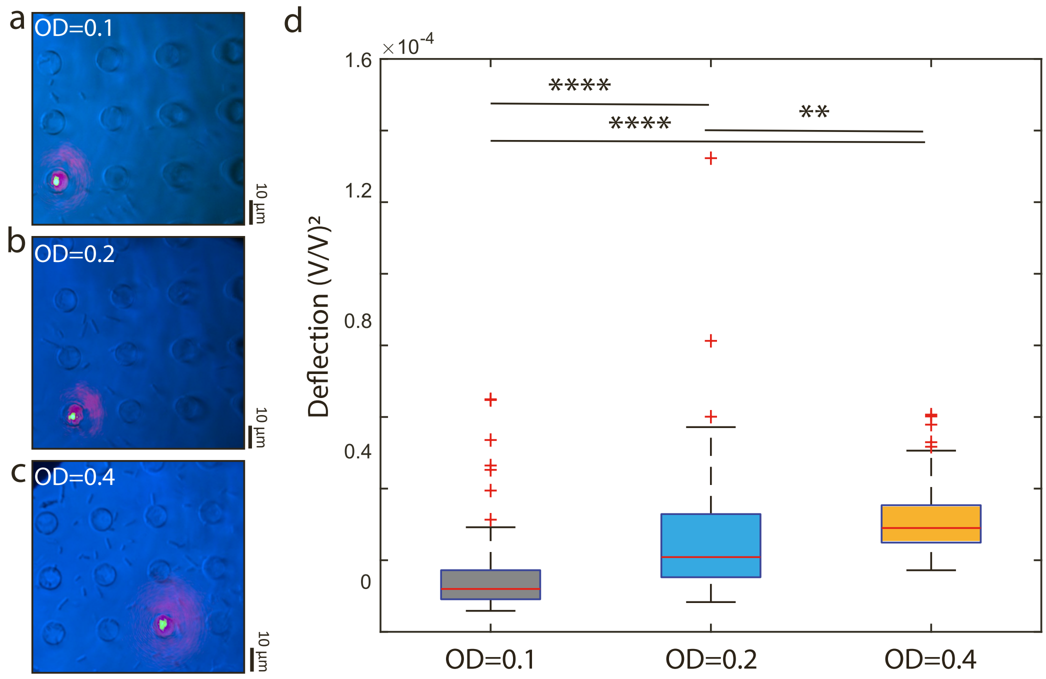

FIG. S4. Comparison between the variance of the optical signal as the function of hypermotile 7740 *E.coli* cell density in liquid, related to Figure 2. a) Optical image of microwells with OD=0.1 bacterial density. b) Optical image of microwells with OD=0.2 bacterial density. c) Optical image of microwells with OD=0.4 bacterial density. d) Signal variance vs sample optical density (OD=0.1, light grey, ($n = 75$), OD=0.2, light blue, ($n = 75$), and OD=0.4, orange, ($n = 75$). Significant difference was observed for the signal ($p < 10^{-3}$, **; $p < 10^{-6}$, ****). Red horizontal line represents the median values. Measurements are compared using a two-tailed Wilcox ranksum test.

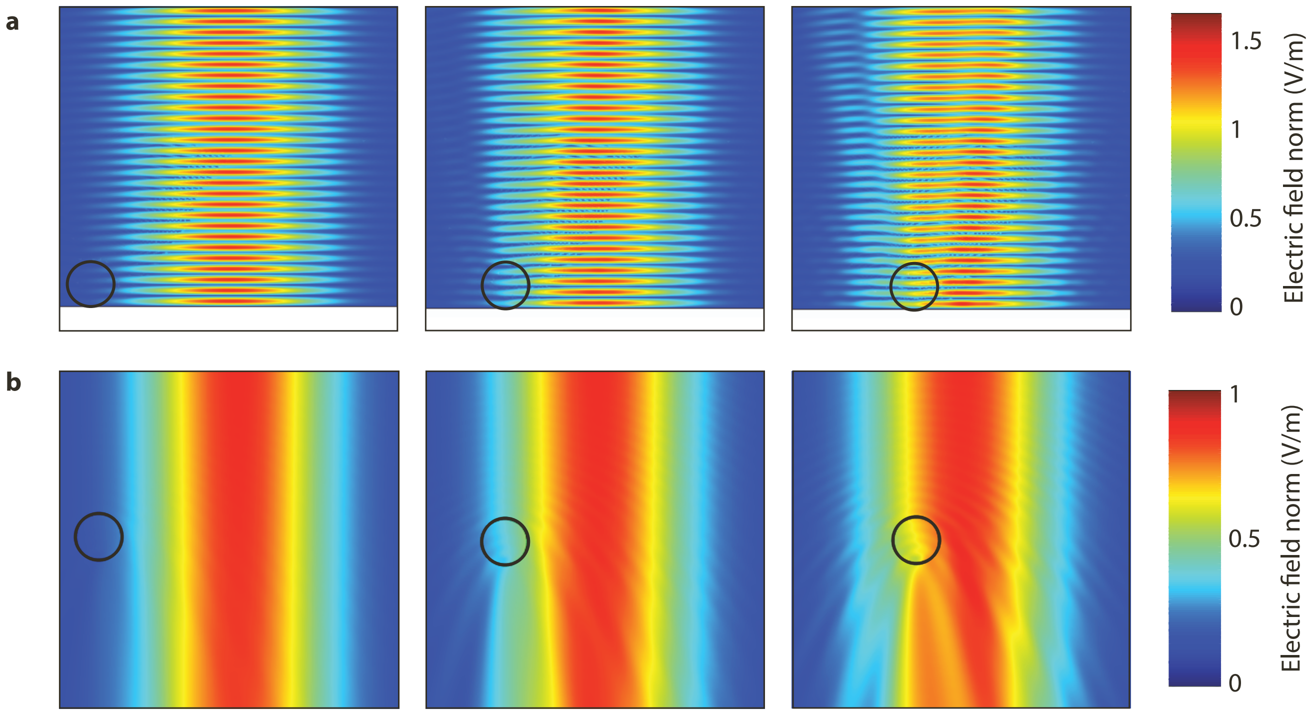

FIG. S5. Simulation of a circular bacterium at varying lateral position with respect to a tightly focused laser beam, related to Figure 3. a) Electric field norm when the bacterium is on the substrate. b) Electric field norm when the bacterium is away from the substrate. For each case , the position of the bacterium with respect to the centre of the beam is shown for $x = -3\mu$m (left),$x = -2\mu$m (centre), and $x = -1\mu$m (right).

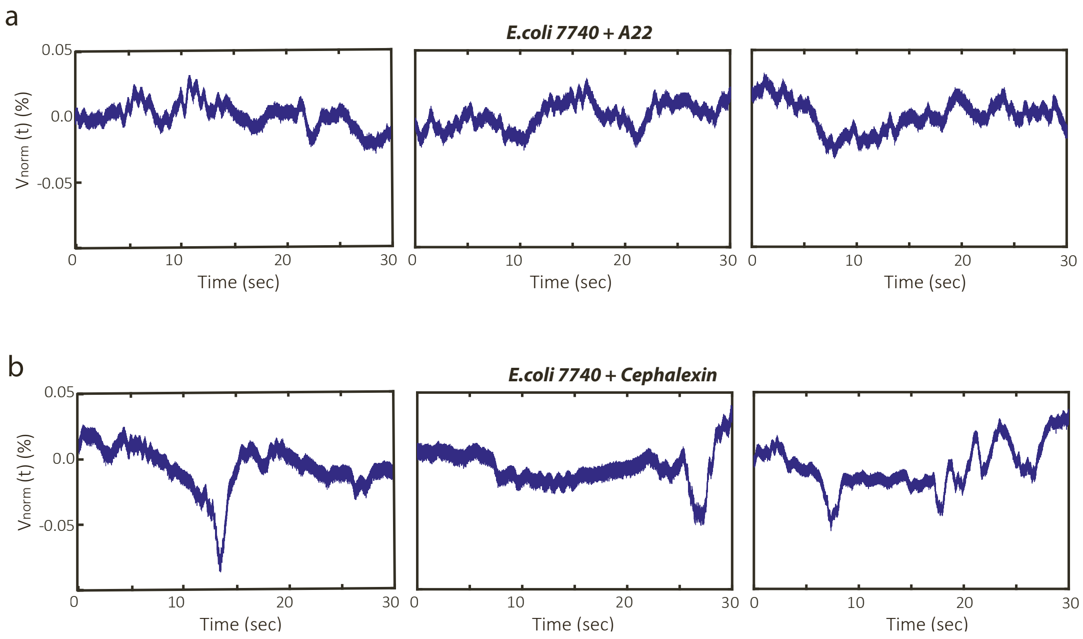

FIG. S6. Measurements on enlarged and elongated cells, related to Figure 3. a) Signals obtained for Cephalexin elongated cells. b) Signals obtained on A22 enlarged cells.

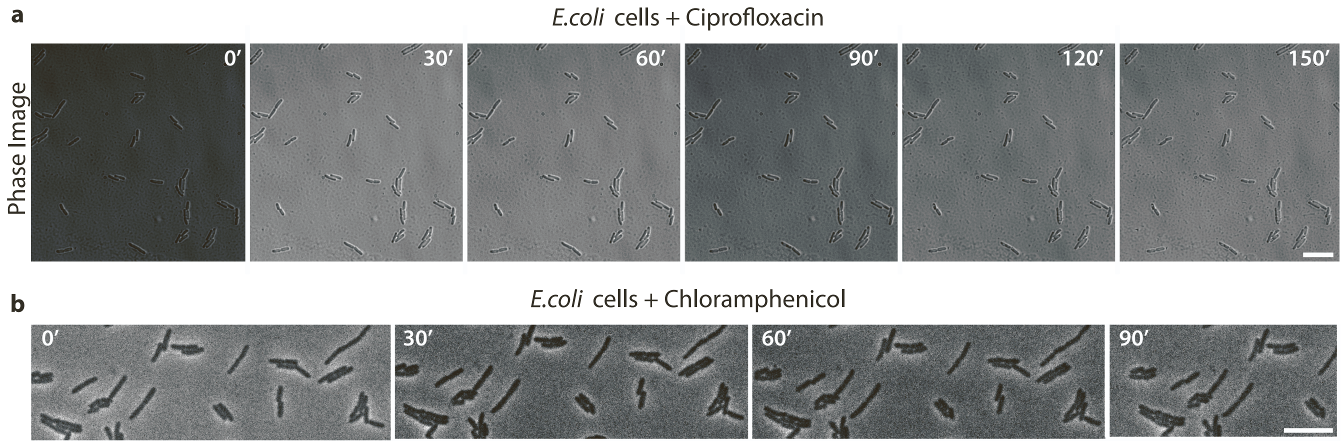

FIG. S7. Phase contrast images of cells grown on LB under agarose pads,related to Figure 3. a) After exposure to Ciprofloxacin. b) After exposure to Chloramphenicol. Time indicated in minutes. Scale bars, 10 microns.

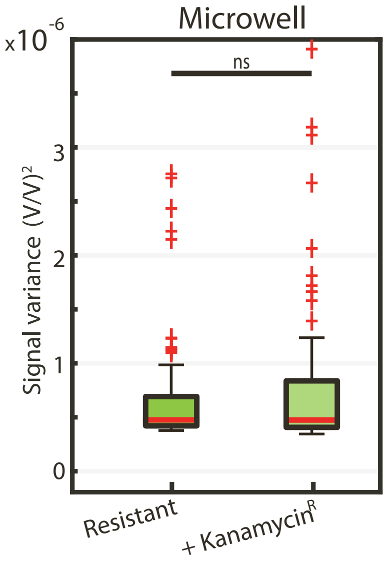

FIG. S8. Signals before and one hour after administering kanamycin (25μg/ml final concentration) to MG1655(*kanR*) resistant cells, related to Figure 4. There is no signal drop observed for kanamycin resistant strains ((light green, (n = 84)(p = 0.53, ns)). Experiments were performed on *E.coli* cells with a chromosomal *KanR* resistance gene exposed to Kanamycin. As it can be observed, no change in the signal variance is seen after exposure to antibiotic, showing that the platform can potentially be used for fast detection of antibiotic resistance with single cell resolution.

TABLE S1. List of antibiotic susceptibility measurements using the present optical method and disk diffusion (EUCAST standard), related to Figure 5.

	Method		
Antibiotic	Nanomotion	Disk Diffusion	Agreement
E.coli (7740)			
Mero	S	S	Yes
Kan	S	S	Yes
Cipro	S	S	Yes
S. enteritidis (S1400)			
Mero	S	S	Yes
P. Aeruginosa (L1262)			
Kan	S	S	Yes

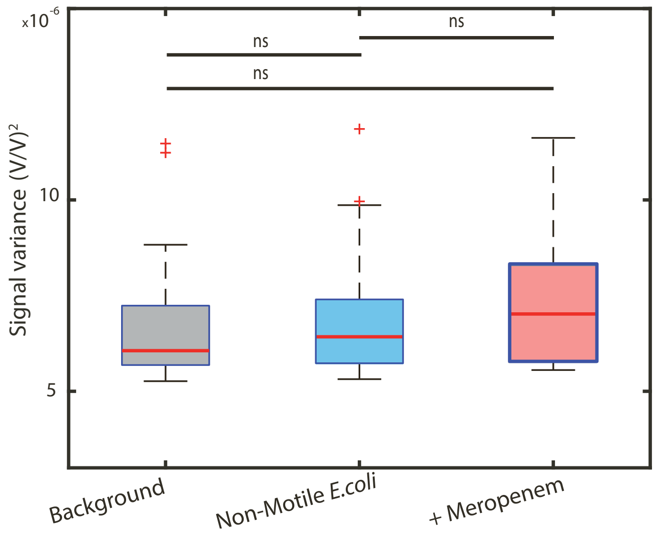

FIG. S9. AST on *E.coli motAB* non-motile cells (cells were lacking motA and motB genes), related to Figure 5. Signals were acquired before and 1.5 hour after administering meropenem (50μg/ml final concentration). There is no statistical difference between the signal on empty wells ((light grey, ($n = 41$), as well as on non-motile ((light blue, ($n = 71$) or antibiotic treated cells ((light red, ($n = 82$). We used a rank sum test for comparison between the conditions, with the following convention ns: $0.05 < \mathrm{p}$.

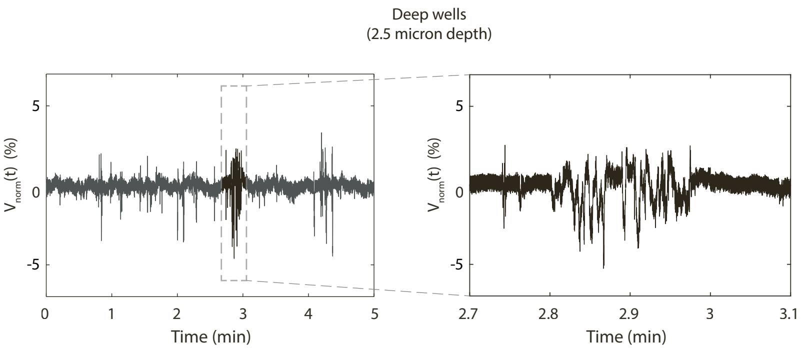

FIG. S10. Signal recorded on deep microwells, related to Figure 1. The signal was recorded for 5 minutes on a 2.5 micron deep well. A trapping event is indicated on the left panel, and the signal is shown in higher detail on the right.

SI REFERENCES

1. Tinevez, J.Y., Perry, N., Schindelin, J., Hoopes, G.M., Reynolds, G.D., Laplantine, E., Bednarek, S.Y., Shorte, S.L., and Eliceiri, K.W. (2017). TrackMate: An open and extensible platform for single-particle tracking. Methods, 115, 80-90.
